# CAXII inhibitors: Potential sensitizers for immune checkpoint inhibitors in HCC treatment

**DOI:** 10.3389/fimmu.2023.1052657

**Published:** 2023-03-16

**Authors:** Rui Han, Jiayin Li, Jing Hony, Zhiwei Xiao, Jinghui wang, Man Yao, Shufang Liang, Lingeng Lu

**Affiliations:** ^1^Department of Chinese Medicine Oncology, The First Affiliated Hospital of Naval Medical University, Shanghai, China; ^2^Department of Chinese Medicine, Naval Medical University, Shanghai, China; ^3^Department of Chronic Disease Epidemiology, Yale School of Public Health, New Haven, CT, United States; ^4^School of Medicine, Center for Biomedical Data Science, New Haven, CT, United States; ^5^Yale Cancer Center, Yale University, New Haven, CT, United States; ^6^Department of Oncology, The First Hospital Affiliated to Guangzhou University of Chinese Medicine, Guangzhou, China; ^7^Department of Oncology, The First Hospital Affiliated to Guizhou University of Chinese Medicine, Guiyang, China

**Keywords:** CAXII inhibition, sensitizer, antitumor immunity, ICIs based therapy, hepatocellular carcinoma, Tiliroside, synergistic effect

## Abstract

Hepatocellular carcinoma (HCC) is a lethal malignancy with a lack of effective treatments particularly for the disease at an advanced stage. Even though immune checkpoint inhibitors (ICIs) have made great progress in the treatment of HCC, durable and ideal clinical benefits still cannot be achieved in plenty of patients with HCC. Therefore, novel and refined ICI-based combination therapies are still needed to enhance the therapeutic effect. The latest study has reported that the carbonic anhydrase XII inhibitor (CAXIIi), a novel type of anticancer drug, can modify the tumor immunosuppression microenvironment by affecting hypoxic/acidic metabolism and alter the functions of monocytes and macrophages by regulating the expression of C-C motif chemokine ligand 8 (CCL8). These observations shine a light on improving programmed cell death protein 1 (PD-1)/programmed cell death ligand-1 (PD-L1) immunotherapy in combination with CAXIIis. This mini-review aims to ignite enthusiasm to explore the potential application of CAXIIis in combination with immunotherapy for HCC.

## Introduction

Hepatocellular carcinoma (HCC) is the most common histological type in primary liver cancer, which occurs predominantly in individuals with chronic liver disease or cirrhosis. HCC is the fourth leading cause of cancer-related death worldwide, and its incidence has been rising globally over the last 20 years (1, 2). It has been expected to keep increasing until 2030 in some countries including the United States, and it has become the fourth of the most common malignancies in China (3, 4). Patients with early-stage HCC may be curable by receiving radical treatment, such as local ablation, surgical resection, or liver transplantation. However, a high recurrence rate still exists (5-year survival rate after surgery is only approximately 35%) (2). In addition, over one-half of HCC patients have already been at an advanced stage when diagnosed due to the lack of sensitive and specific diagnostic tools in the clinic. More importantly, the options of available therapeutic strategies for those patients are also limited (5). For instance, a multikinase inhibitor (MKI), a newly developed anticancer agent compared to traditional chemotherapy, has already made significant progress in HCC treatment in recent years. MKIs such as lenvatinib, cabozantinib, and regorafenib have been approved for treating advanced or metastatic HCC (6–8). However, a considerable number of HCC patients are still unable to gain durable and ideal clinical benefits (7–10).

Since most HCCs are derived from chronic inflammatory liver damage (e.g., hepatitis B virus-related) (11, 12), such a disease is considered an inflammation-related cancer. Therefore, HCC patients are theoretically considered to benefit from immunotherapy (5). In addition, notable advances in the comprehension of HCC immunogenicity have been achieved over the last few years, leading to the evaluation of immune checkpoint inhibitors (ICIs) as a frontline treatment in this setting. However, based on the results of clinical trials, ICI monotherapy has been found to provide limited efficacy against HCC, with the treatment being beneficial in a limited cohort of patients (13, 14). Additionally, the role of ICIs in combination with other anticancer agents (including MKIs) in unresectable treatment-naive HCC has also been assessed in some phase I–III clinical trials (**Table 1**) (6, 14, 18–21). For instance, in the IMbrave150 trial (phase III study), which randomized HCC patients to atezolizumab plus bevacizumab or to sorafenib monotherapy, the overall survival (OS) and independent review facility-assessed progression-free survival (PFS) both were superior in patients receiving the immunotherapy-based combination compared to those of the monotherapy group (22). Furthermore, as reported in the phase III HIMALAYA trial, the risk of death for HCC patients in the durvalumab plus tremelimumab group was significantly lower than that of the sorafenib monotherapy group (**Table 1**) (16). Such evidence not only suggests a novel standard of care in HCC patients but also proves the superiority of ICI-combined therapy. However, several unanswered questions remain in such settings, including the lack of biomarkers predictive of response to immunotherapy and the presence of a non-negligible proportion of patients who do not gain benefit from ICIs (21, 23, 24). Therefore, how to explore a novel and effective ICI-based combination therapeutic strategy to obtain better clinical benefits has become a hot and difficult issue in the current international frontier of HCC treatment (25–29).

**Table 1:** Selected trials of ICI-combined therapies for HCC.

HCC stage	Therapeutic regimen	Study	Phase	Total patient number	ORR (RECIST1.1)	Median PFS (months)	Median OS (months)	Line of therapy
Advanced HCC (not eligible for surgical and/or locoregional tderapies or progressive disease after surgical and/or locoregional tderapies)	Nivolumab/ipilimumab	NCT01658878 (CheckMate 040) (15)	I/II	148	32% (95% CI, 20-47) vs. 27 (95% CI, 15-41)	NA	22.8 (95% CI, 9.4-not reached) vs. 12.5 (95% CI, 7.6-16.4)	First-line
Advanced HCC (not eligible for locoregional tderapy)	Tremelimumab/ durvalumab	NCT03298451 (HIMALAYA) (16)	III	1171	20.1% (95% CI, NA) vs. 5.1% (95% CI, NA)	3.8 (95% CI, 3.7–5.3) vs. 4.1 (95% CI, 3.8-5.5)	16.4 (95% CI, 14.2–19.6) vs. 13.8 (95% CI, 12.3-16.1)	First-line
Advanced HCC [not amenable to a curative treatment approach (e.g., transplant, surgery, ablation tderapy) or locoregional tderapy (e.g., TACE)]	Cabozantinib/ Atezolizumab (vs. sorafenib)	NCT03755791 (COSMIC-312) (17)	III	837	13% (95% CI, 8.9-17.6) vs. 5% (95% CI, 1.8-10.4)	6.8 (99% CI, 5.6-8.3) vs. 4.2 (99% CI, 2.8-7.0) (HR 0.63; 99% CI, 0.44-0.91; P = 0.0012)	15.4 (96% CI, 13.7-17.7) vs. 15.5 (96% CI, 12.1-NE) (HR 0.90; 96% CI, 0.69-1.18; P=0.44)	First-line
Unresectable HCC (immunotderapy-naive; have eitder progressed on, are intolerant to, or refused treatment witd sorafenib or anotder approved TKI)	Tremelimumab +durvalumab	NCT02519348 (18)	I/II	332	24.0% (95% CI, 14.9-35.5) vs. 10.6% (95% CI, 5.4-18.1)	2.2 (95% CI, 1.9-5.5) vs. 2.1 (95% CI, 1.8-3.4)	18.7 (95% CI, 10.8–28.3) vs. 13.6 (95% CI, 8.7-17.6)	Subsequent-line
Advanced or metastatic and/or unresectable HCC (not amenable to a curative approach)	Atezolizumab/ Bevacizumab (vs. atezolizumab) (group F)	NCT02715531 (GO30140) (19)	Ib	119	20% (95% CI, 11-32) vs. 17% (95% CI, 8-29)	5.6 (95% CI, 3.6-7.4) vs. 3.4 (95% CI, 1.9-5.2) (HR 0.55; 80% CI, 0.40-0.74; P=0.011)	NE	First-line
Unresectable HCC (HCC for which no otder appropriate tderapy is available)	Lenvatinib+ pembrolizumab	NCT03006926 (KEYNOTE-524) (20)	Ib	104	36.0% (95% CI, 26.6-46.2)	8.6 (95% CI, 7.1-9.7)	22 (95% CI, 20.4-NE)	First-line
Locally advanced or metastatic and/or unresectable HCC	Atezolizumab/ Bevacizumab (vs. sorafenib)	NCT03434379 (IMbrave150) (21)	III	501	27.3% (95% CI, 22.5-32.5) vs. 11.9 (95% CI, 7.4-18.0)	6.9 (95% CI, 5.7-8.6) vs. 4.3 (95% CI, 4.0-5.6) (HR 0.65;95% CI, 0.53-0.81; P<0.001)	19.2 (95% CI, 17.0-23.7) vs. 13.4 (95% CI, 11.4-16.9) (HR 0.66; 95% CI, 0.52-0.85; P<0.001)	First-line

CI, confidence interval; HR, hazard ratio; NA, not available; NE, not estimable; NR, not reached; ORR, objective response rate; OS, overall survival; PFS, progression-free survival; RECIST 1.1, Response Evaluation Criteria in Solid Tumors 1.1; ICI, immune checkpoint inhibitor; HCC, hepatocellular carcinoma.

## Exsiting carbonic anhydrase XII inhibitor in solid cancer treatment

Carbonic anhydrase XII (CAXII) is a transmembrane zinc metalloenzyme involved in the regulation of the tumor microenvironment, contributing to tumor cell proliferation, invasion, migration, and pluripotency (30). It has been reported that CAXII is overexpressed in HCC, and its level is significantly negatively correlated with the prognosis of HCC patients, which may act as an independent prognostic factor (31–33).

A highly hypoxic tumor microenvironment is a hallmark for human cancer including HCC, which results from the Warburg effect and the surrounding fibrotic tissues caused by persistent chronic inflammation. The overproduction of pyruvate, lactate, and carbonic acid in response to hypoxia aggravates the hypoxic/acidic microenvironment, leading to the enhancement of tumor invasion, tumor immune surveillance escape, and local inflammation. As a regulator of hypoxic stress and acidity, CAXII can affect the tumor microenvironment by regulating proteins such as 14V-ATPase and 15V-ATPase, thereby promoting HCC progression (32, 33). Therefore, a CAXII inhibitor (CAXIIi) is thought to be a novel anti-HCC agent, controlling HCC progression and reducing immunosuppressive stress *via* the regulation of hypoxic/acidic metabolism. However, the investigation of the antitumor effects and mechanisms of CAXIIis is still in fragmentation. The development and exploration of antitumor CAXIIis have become a new global research hot spot (30, 32, 33).

**Figure 1:**
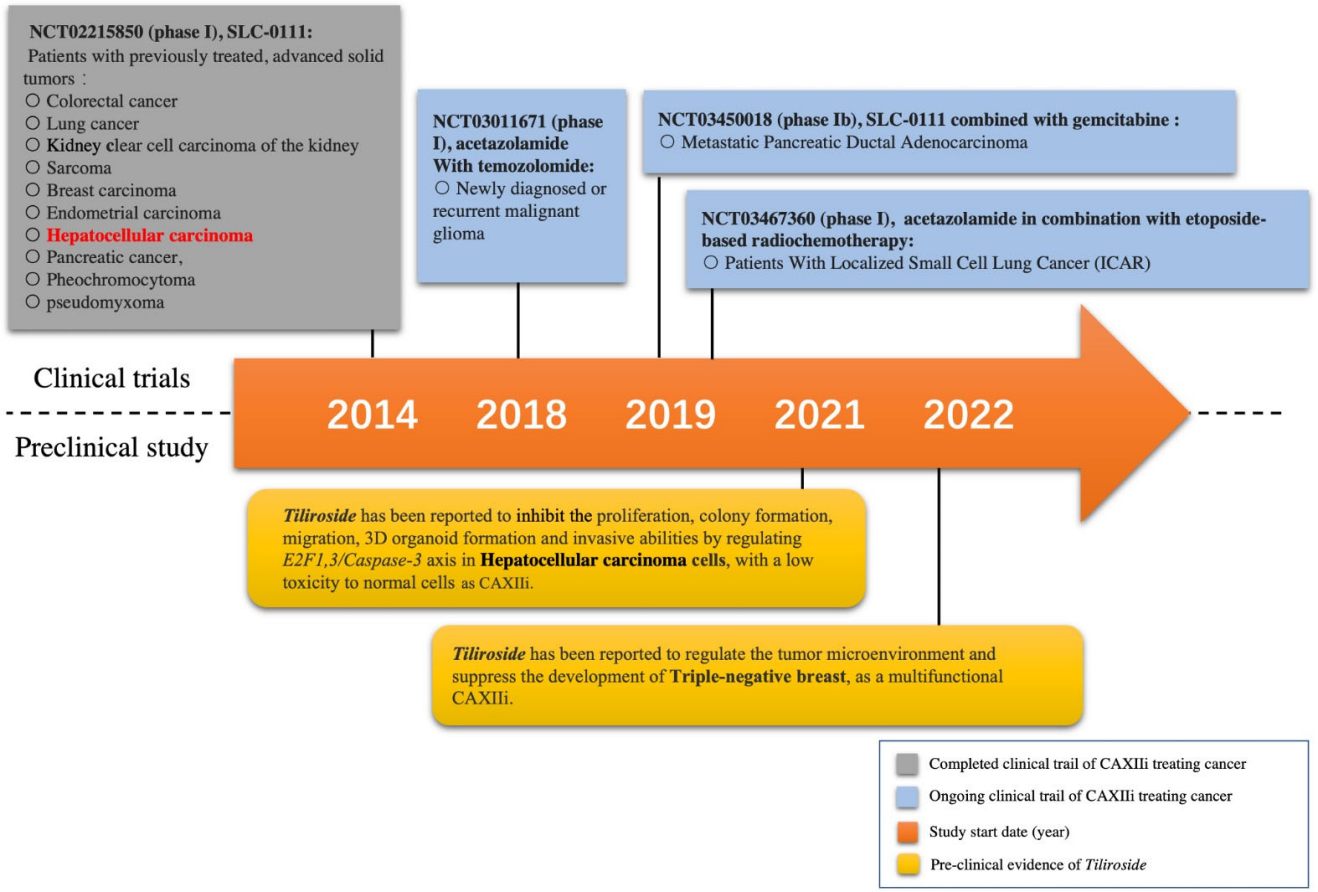
Milestone of selected CAXII inhibitor (CAXIIi) research and development. Currently, only one CAXIIi has been tested in a clinical trial for hepatocellular carcinoma treatment. *Tiliroside* monotherapy has been found to inhibit the development of hepatocellular carcinoma cells by a preclinical study.

The initiation of CAXIIis on cancer treatment has started recently. A phase I clinical trial of the first highly selective small-molecule inhibitor of both CAIX and CAXII (SLC-0111) recently has been completed with promising results (34). Briefly, 17 patients with 10 different cancer types including one HCC patient were recruited to be on the inhibitor. The results showed that the inhibitor was safe in patients with previously treated advanced solid tumors (**Figure 1**) (34). Moreover, a multicenter, open-label, phase Ib study of SLC-0111 in combination with gemcitabine for metastatic pancreatic ductal cancer in subjects positive for CAIX has been conducted since 2018 (**Figure 1**). Acetazolamide, as a multiple carbonic anhydrase inhibitor (including CAXII), and its combination with radiochemotherapy have been tested in lung cancer (NCT03467360), while the combination of acetazolamide and temozolomide has also been trialed for brain cancer (NCT03011671) (**Figure 1**) (35, 36). To our knowledge, no other attempt has been made to investigate the therapeutic effect of CAXIIis in HCC treatment so far, which is still a desertlike field.

## Enhance anti-tumor immunity by modulating macrophage

Macrophages, some of the most abundant immune cells in tissues, are highly heterogeneous and can switch between different functions in the context of their niches where they are located. Their functions are determined by their polarized types (M1 or M2). Generally, the M1 subtype secretes inflammatory cytokines and reactive oxygen intermediates and presents antigen to tumor-suppressive T cells, stimulating the immune response (37, 38). In contrast, the M2 subtype is a tumor-promoting macrophage, inducing T-cell anergy (or exhaustion) and angiogenesis, producing extracellular matrix components, and repairing damaged tissues (37, 38). Although the origins of macrophages in many cancers remain uncertain, most of the macrophages recruited to the tumor microenvironment, known as the tumor-associated macrophages (TAMs), are the tumor-supportive M2 subtype (39). Abundant M2 macrophages were positively associated with poor survival in patients with breast cancer (40, 41). Recent studies have found that effectively interfering with macrophages is a potential strategy to treat cancer (42–44).

Evidence has shown that the metabolic reprogramming of macrophages can eventually inhibit tumor growth by regulating T cells (45). With tumors developing, the response protein of protein kinase R (PKR)-like endoplasmic reticulum kinase (PERK) is produced by the interaction between macrophages and cancer cells, which participates in the remodeling of several key metabolic pathways of macrophages. Blocking the expression of PERK can inhibit the downstream metabolic signaling in tumor-infiltrating macrophages, resulting in more effector T cells to fight the cancer cells and consequently enhancing the efficacy of PD-1 inhibitors (45). Therefore, targeting or editing the metabolism of macrophages has been thought to be a novel therapeutic treatment in combination with PD-1 inhibitors. Furthermore, it has been reported recently that CAXIIis can interfere with the metabolism of macrophages by regulating *CCL8* and *PERK* expression (46) and promote the therapeutic effect of PD-1 inhibitors in HCC treatment (47).

## Effects of carbonic anhydrase XII inhibitor on macrophages

Evidence has shown that *CAXII* was the most significantly upregulated gene among all αCA family genes in tumor-infiltrating monocytes when comparing to the ones in the paired non-tumor liver tissues (45). Moreover, the expression level of *CAXII* mRNA increased in tumor-infiltrating monocytes but not in other CD14+ cell components in both tumor tissue and non-tumor liver tissue, indicating that *CAXII* might contribute to HCC progression (45). It was also shown that a positive correlation existed between the expression level of *CAXII* and glucose transporter GLUT1 in tumor-purified CD14+ cells, which may affect the glycolytic switch in tumor-infiltrating monocytes and macrophages (45).

In addition, the glycolysis inhibitor 2-deoxyglucose (2-DG) or 6-phosphofructo-2-kinase/fructose-2,6-biphosphatase 3 (PFKFB3, a key glycolytic enzyme) can effectively reduce the expression of *CAXII* mRNA and protein levels in HepG2 tumor culture supernatant (TSN)-treated monocytes (peripheral blood purified CD14+ cells from healthy subjects) (45, 46). Meanwhile, the tumor-triggered glycolytic switch in monocytes has been found to induce the activation of hypoxia-inducible factor (HIF)1α and the production of tumor necrosis factor (TNF)-α, interleukin (IL)-10, and IL-1β, which in turn synergistically upregulates the *CAXII* expression in monocytes (45, 46). Therefore, it has been considered that aerobic glycolysis can induce the *CAXII* upregulation through HIF1α and the autocrine cytokine-dependent pathways in monocytes and macrophages, and CAXII was also found to mediate the survival of macrophages and monocytes in an acidic microenvironment in HepG2 cells (45).

The C-C motif chemokine ligand 8 (CCL8), a member of the CC chemotactic protein family, can recruit monocytes, T cells, eosinophils, basophils, natural killer (NK) cells, and dendritic cells by binding to the receptors of CCR1, CCR2, CCR3, and CCR5. It acts as an important immune regulator in inflammatory response, antitumor immunity, and acute graft-versus-host disease (aGVHD) (48). Evidence has shown that the levels of matrix metalloproteinase (MMP)9, vascular endothelial growth factor A (VEGFA), and CCL8 are all increased in tumor monocytes, with CCL8 showing the most pronounced upregulation compared to non-tumor monocytes. It has been demonstrated that the glycolysis-induced upregulation of *CAXII* expression was related to the CCL8 production in tumor-associated monocytes and macrophages (47, 48). Moreover, CCL8, as a CC chemokine that utilizes multiple cellular receptors to attract and activate human leukocytes, was reported to significantly promote the migration of HepG2 cells and increase the expression levels of *vimentin (VIM)* and *SNAI1*, two epithelial–mesenchymal transition (EMT)-related markers (48). The mRNA level of *CCL8* in tumor-infiltrating monocytes was also found to be positively correlated with *VIM*, negatively with cadherin 1 (*CDH1*), and positively with the metastatic potential of HCC. Therefore, the *CAXII/CCL8* axis has been thought to be involved in the progression and metastasis of tumor and a potential therapeutic target (47, 48).

Further evidence displayed that CAXIIis significantly increased the therapeutic effects (including suppressing tumor growth, attenuating tumor metastasis, and enhancing OS of mice) of anti–PD-1 antibodies on HCC compared to either single CAXII inhibitor group or single anti–PD-1 antibody group alone (P < 0.05) *in vivo*, respectively (47). In addition, CAXIIis have also been found to increase the apoptosis of macrophages, reduce the ratio of macrophages in total CD45+ cells, and increase the ratio of CD8+ T cells in total tumor lymphocytes (47). Such a result is partly consistent with our previous study in which targeting CAXII can effectively inhibit the development of liver cancer and triple-negative breast cancer cells (32, 49). What is noteworthy is that even though CAXII was mainly concentrated on tumor-infiltrating macrophages in the majority of tumor samples, it has also been found that CAXII might be important for the survival and function of M2-subtype macrophages in the tissues of HCC (47). Overexpression of CAXII may mediate the accumulation of M2 cells in tumor tissues *via* regulating PERK and CCL8 (47, 50, 51).

## Potential carbonic anhydrase XII inhibitors

Since the immunoregulation ability of CAXIIis has been revealed, it has become a crucial issue to choose an appropriate CAXIIi that can properly synergize with the therapeutic effect of ICIs. In addition, some CAXIIis have already displayed excellent anticancer effects on different types of cancer cells (32, 34). The CAXII inhibitor SLC-0111 has been found to enhance the cisplatin antitumor activity and suppress the growth and invasion of head and neck squamous cell carcinoma (52). Meanwhile, it has also been found to sensitize patients with either melanoma or breast cancer to PD-1/PD-L1 inhibitors by enhancing the Th-1 response (53). In addition, the therapeutic strategy of acetazolamide combined with radiotherapy has been tested in lung cancer (NCT03467360), and the combination of acetazolamide plus temozolomide has also been trialed for brain cancer (NCT03011671) (**Figure 1**) (35, 36). However, few studies have been conducted to show the combination of CAXIIis with PD-1/PD-L1 ICIs in HCC thus far. It would be more intriguing if the tumor-suppressive effect of CAXIIis could be further enhanced by combining with PD-1 inhibitors.

The plant *Tribulus terrestris* L. (TT) can be found in many regions of Asia and Africa and has been used in traditional Chinese medicine and Ayurvedic medicine as an herb to treat liver diseases for thousands of years. ***Tiliroside*** (*TS*), one of the main extractions from this herb (54, 55), has been found to possess anti-inflammatory, anticholinesterase, and antioxidant activities (56). Moreover, our recent study has further revealed its multiple anticancer effects on HCC cells and, more importantly, its property of CAXIIi (32). Evidence has displayed that *TS* can inhibit the proliferation, colony formation, migration, three-dimensional (3D) organoid formation, and invasive abilities by regulating apoptosis and stemness in HCC cells while having low toxicity to normal cells (32). The study also found that *TS* can regulate the tumor microenvironment by modulating the levels of pHi, pHe, and lactate, therefore inhibiting the development of triple-negative breast cells, and act as a multifunctional CAXIIi (**Figure 1**) (49).

**Figure 2:**
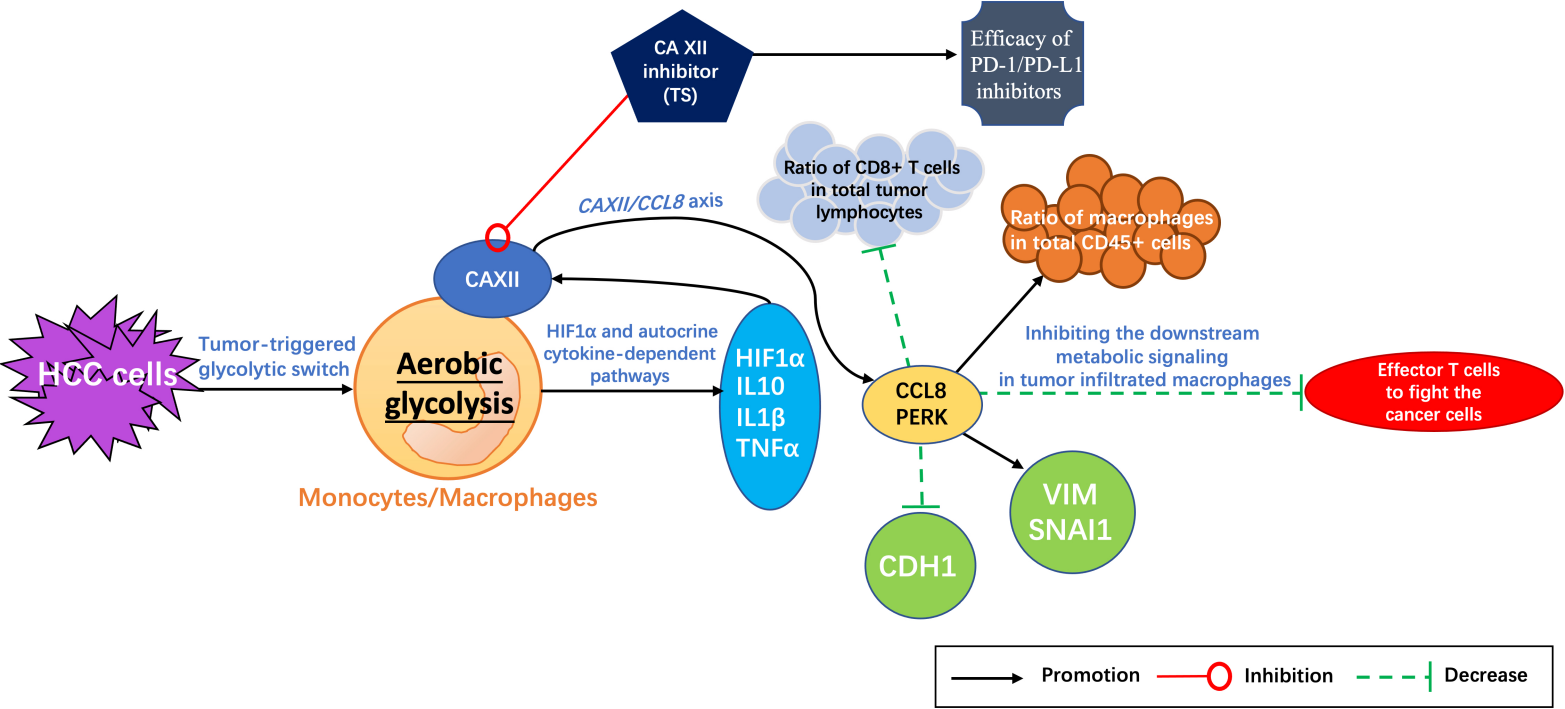
Potential mechanism of *tiliroside (TS)* enhancing the therapeutic effect of PD-1/PD-L1 inhibitors. HCC cells trigger the metabolic switch from oxidative phosphorylation to aerobic glycolysis in tumor-infiltrating monocytes and macrophages. The activation of HIF1α is induced and consequently the cytokines of TNF-α, IL-10, and IL-1β are produced, which in turn synergistically upregulate the CAXII expression in monocytes and macrophages, and then increase the expression of protein kinase R (PKR)-like endoplasmic reticulum kinase (PERK). CAXII inhibitors (such as *TS*) can regulate the infiltration of lymphocytes (reducing the ratio of macrophages in total CD45+ cells and increasing the ratio of CD8+ T cells in total tumor lymphocytes) and suppress the expression of vimentin (VIM) and SNAI1 but increase CDH1 by regulating the expression of C-C motif chemokine ligand 8 (CCL8) and PERK, consequently increasing the therapeutic effect of PD-1/PD-L1 inhibitors. HCC, hepatocellular carcinoma; HIF1α, hypoxia-inducible factor (HIF)1α; TNF-α, Tumour necrosis factor α; IL-10, Interleukin 10; IL-1*β, Interleukin-1β; CAXII, carbonic anhydrase XII; SNAI1, Snail Family Transcriptional Repressor 1; CDH1, cadherin 1; PERK, Protein kinase RNA-like endoplasmic reticulum kinase.

Therefore, *TS* is a promising candidate in combination with PD-1 inhibitors to improve the immunotherapy efficacy (**Figure 2**).

## Side effects of CAXIIi application

At present, there are a few studies on the safety of CAXIIis. For instance, a novel CAXIIi named 6A10-Fab-fragment is currently being trialed in a phase I study to evaluate its maximum tolerated dose and patient-specific dosimetry in a combined therapeutic strategy for patients with glioblastoma (NCT05533242) (57). Moreover, for treating advanced solid tumors, the safety and tolerability of a highly selective small-molecule inhibitor of CAIX/CAXII (SLC-0111) have already been evaluated by a phase I study (NCT02215850) with very promising results (34). For details, as reported by this study, SLC-0111 was generally well tolerated at doses of 1,000 mg daily or below, but frequent early discontinuation was observed at doses of 2,000 mg. However, as displayed by pharmacokinetic (PK) assessments, both the 1,000- and 2,000-mg doses gained similar levels of drug exposure following single doses of SLC-0111. Moreover, majority of the reported drug-related adverse events (AEs) were grade 1 or 2 in severity, and the most frequent AEs were fatigue, nausea, anorexia, diarrhea, and vomiting, all of which were reversible (34). Only one patient has been reported to undergo a grade 3 hepatobiliary disorder, which was considered to be a serious AE related to SLC-0111 but not a dose-limiting toxicity (DLT) (34). Furthermore, the toxicities of SLC-0111 will be continually evaluated by other ongoing clinical trials, such as NCT03450018. Apparently, the safety evaluation of CAXIIis still requires more research investment.

## Future directions

Cancer is not a simple disease but a complex product of changes in the genome and the body’s internal environmental response. Therefore, the current cancer immunotherapy targeting a single target, such as PD-1/PD-L1 inhibitor, Cytotoxic T-lymphocyte Antigen-4 (CTLA-4) inhibitor, and Chimeric antigen receptor (CAR) T-cell therapy (CAR-T), has very limited therapeutic effect on most cancers (58, 59). Thus, new combination therapies have been sought to improve clinical benefits. As mentioned above, CAXIIis, novel and strong anticancer agents, can enhance the therapeutic effect of PD-1 inhibitors by regulating the antitumor immunity in HCC (47), providing a new thought for cancer treatment. Therefore, the roles of CAXIIis in the integrated metabolic space of tumor and immune cells should be further explored in a future study for exerting their anticancer and immunomodulatory effects in a more efficient way. On the other hand, based on a more comprehensive and clear regulatory network of CAXIIis, more chances could be gained by expanding their application in different combination therapies with multiple targets for treating cancer, as shown in a preclinical study that combined CAIX and CAXII dual inhibition with immune checkpoint blockade resulting in improved efficacy of immune therapy in melanoma and breast cancer (53). Meanwhile, the effects of CAXIIis combined with different or multiple immune checkpoint blockades should also be evaluated for seeking novel ICI-based treatment (53). Moreover, adding a safe and targeted drug delivery system, which is a pattern of specifically designed carriers, to the application of CAXIIis in cancer treatment is also a recommended potential research direction (60, 61). Those nanoparticles were created to encapsulate and deliver agents to specific lesion sites with enhanced solubility and efficacy of drugs and reduced interaction with untargeted tissues (60, 61). For instance, our previous study has reported the capability of promoting the therapeutic effect, possessed by a novel lipid-based nanoparticle, LNP-DP1, in treating HCC (60). Furthermore, currently, to the best of our knowledge, none of the biomarkers has been reported to be applied in predicting the effectiveness, safety, or toxicity of CAXIIi treatment. Such unsolved issue is like an invisible barrier that might impede the potential application of CAXIIis in cancer treatment, including cancer immunotherapy (62). Therefore, the assessment of biomarkers for CAXIIis is encouraged in a future study. The same future investigation is also required for evaluating the safety and tolerability of CAXIIis.

## Conclusion

Accumulating evidence has demonstrated the anticancer effect of CAXIIis on different types of cancers, including HCC. Therefore, such agents are considered promising novel anti-HCC drugs that can suppress HCC progression (32). Moreover, CAXIIis have been found to reduce the immunosuppressive stress mediated by hypoxic/acidic metabolism, regulate the expression of CCL8, and affect the functions of monocytes and macrophages, thereby improving the antitumor immunity and enhancing the therapeutic effect of PD-1 inhibitors in HCC (47). In summary, CAXIIis are not only effective single anticancer agents but also potential sensitizers of PD-1 inhibitors. Thus, a candidate such as *TL*, a novel CAXIIi with high efficiency against tumors and low toxicity to normal cells, has been considered to hold untapped potential in ICI-based combination therapy for HCC treatment. Furthermore, more promising candidates of CAXIIis are warranted to be included in future studies to explore more effective therapeutic strategies and novel therapeutic targets for HCC. Additionally, the combination setting of PD-1 inhibitors plus CAXIIis and antiangiogenic agents may offer more effective therapeutic options to patients if the function of CAXIIis as a PD-1 inhibitor sensitizer has been fully evaluated.

## Author contributions

RH generated the original concept of this manuscript with the help of LL. JL, JH, ZX, JW, helped to write the paper, MY and SL helped to generate the figure. All authors contributed to the article and approved the submitted version.
